# Inquiry Based Stress Reduction (IBSR) Improves Overall Stuttering Experience among Adults Who Stutter: A Randomized Controlled Trial

**DOI:** 10.3390/jcm10102187

**Published:** 2021-05-18

**Authors:** Omrit Feldman, Eran Goldstien, Benjamin Rolnik, Ariel B. Ganz, Shahar Lev-Ari

**Affiliations:** 1 Department of Health Promotion, School of Public Health, Sackler Faculty of Medicine, Tel-Aviv University, Tel-Aviv 69978, Israel; omritfel@gmail.com (O.F.); erangold1122@gmail.com (E.G.); 2 Department of Genetics, Stanford University, Stanford, CA 94305, USA; abganz@stanford.edu (B.R.); rolnik@stanford.edu (A.B.G.)

**Keywords:** IBSR, stuttering, mindfulness, cognitive reframing, adults who stutter, OASES, treatment

## Abstract

Stuttering is a speech disorder that can cause disturbances in the timing and flow of speech. In addition to being a communication disorder, stuttering is often accompanied by a reduction in the quality of life and has impacts on social status, mental well-being, self-acceptance, and the chances of integration into the labor market. The Inquiry Based Stress Reduction (IBSR) program, developed in the United States by Byron Katie in 1986, is the clinical application of “The Work” method (Thework.com) and represents an emerging mindfulness and cognitive-reframing method. IBSR has been demonstrated to improve mental health and well-being in adults and may alleviate psychological and psychosocial symptoms of stuttering. The purpose of this trial was to examine the effect of a 12-week IBSR intervention on the overall stuttering experience and indicators of anxiety, psychological flexibility, and well-being among adults who stutter (AWS). This study was a randomized controlled clinical trial. Participants were randomized to IBSR (n = 28) and control (n = 28) groups. Validated questionnaires of overall stuttering experience (OASES-A), anxiety (STAI), psychological flexibility (PFQ), and satisfaction with life (SWLS) were completed before, after, and one month after the intervention. An intention-to-treat approach was implemented for analysis. Our results show that participants in the IBSR intervention group exhibited a greater improvement in their overall stuttering experience as compared to the control group, as well as in general information on stuttering awareness and perception, reactions to stuttering, communication in daily situations, and quality of life. In addition, we found a greater reduction in anxiety levels and an increase in satisfaction-with-life scores in the IBSR group. These results indicate that IBSR can improve the overall stuttering experience.

## 1. Introduction

Stuttering is a speech disorder that can cause disturbances in the timing and flow of speech [1]. In addition to communication difficulties, stuttering is also associated with a decline in the quality of life of adults who stutter (AWS) [2] and has impacts on social status [3], mental well-being [4], self-acceptance [5], and the chances of integration into the labor market [6]. AWS may have to cope on a daily basis with emotional struggles during stuttering, as well as shyness and social-avoidance behaviours that result from fear of coping with communication in certain social situations, and high levels of chronic anxiety, particularly social anxiety [3,7,8]. 

Stuttering is routinely treated directly by a speech therapist (“direct” treatment); however, according to the literature, this kind of treatment is less effective as an exclusive treatment and may be beneficially combined with "indirect" mental-healthcare treatments to achieve optimal results [9,10,11]. Indirect speech treatments focus on the emotional–social aspects of stuttering, with the aim of conferring abilities and resources that will enable AWS to cope with the problems and ameliorate the associated psychological and social symptoms [9,11,12,13,14,15,16].

Meditative practices have been found to improve quality of life, mood, and reduce stress, anxiety, and depression among people who stutter [1,17,18,19]. In recent years, specific mind–body interventions have been examined as treatments to support people who stutter, including Acceptance and Commitment Therapy (ACT) [20,21,22], Cognitive Behavioral Therapy (CBT) [14,22,23], and Mindfulness-Based Stress Reduction (MBSR) [24,25,26,27]. 

The Overall Assessment of the Speaker’s Experience of Stuttering (OASES) instrument was developed to document the impact of stuttering on a person’s life [28]. OASES is currently the gold standard for assessing general information about speech, reactions to stuttering, the level of daily communication, and the quality of life of people who stutter [28,29]. The practice of meditation is expected to cause AWS to feel less stressed [30], experience a greater sense of well-being [22], be less disturbed by the adverse effects associated with their disorder [31], and develop a high more positive picture of reality [32,33]. However, there is a lack of randomized clinical trials to assess effect of meditative practices on OASES levels of adults who stutter. 

One of the latest meditation methods to emerge is Inquiry Based Stress Reduction (IBSR), developed in the United States by Byron Katie in 1986. The IBSR technique is the clinical application of “The Work” (Thework.com) and has already been practiced by hundreds of thousands of people in more than 30 countries around the world [34]. This technique teaches participants to systematically identify the stress and anxiety-provoking thoughts, which are then investigated meditatively in order to experience cognitive reframing and achieve a different interpretation of the reality experienced [33,34,35].

The effectiveness of the IBSR method has been previously tested in a population of breast cancer survivors where it was found to improve psychological and physical metrics as well as functional capabilities upon their return home [36]. Among the general population, the method was found to ameliorate psychological symptoms [37,38]. In addition, the meditation technique of the IBSR method helped reduce burnout from the profession in a population of teachers and promoted mental well-being [39,40].

The aim of the IBSR technique is to enable participants to free themselves from attachment to painful thoughts (hold onto them and not let go of the thoughts) that cause them distress and suffering, and thus, to experience peace, joy and inner freedom as a result of self-exploration of thoughts and beliefs related to stressful situations. Practitioners of the technique identify their negative thoughts and then explore them, with the goal of becoming aware of how the thought causes suffering, as well as deepen awareness of their attachment to it. They are then encouraged to conduct an internal inquiry and investigation into whether the thoughts are true or not [34,36,38]. 

The effectiveness of IBSR has yet to be examined among the AWS population. Therefore, the purpose of this current study was to evaluate the impact of this method on the stuttering experience and psychological indicators of anxiety, psychological flexibility, and satisfaction with life of adults who stutter. 

## 2. Materials and Methods

### 2.1. Study Population and Recruitment Process

Israeli men and women adults who stutter were recruited from the Israel Stuttering Association (AMBI) and from social networks advertisements between October 2017 and April 2018. Study inclusion criteria were diagnosis of either developmental or acquired stuttering, age of 18 years or above, and capability to understand and complete the study outcome instruments and informed consent form. Exclusion criteria were diagnosis of severe mental illness, inability to understand or read Hebrew, and subjects who declared that they could not participate in the whole duration of the trial.

### 2.2. Study Design

The study was a randomized controlled clinical trial. Fifty-six subjects were randomized to one of the two groups: (1) the intervention group—the 28 members of this group participated in an IBSR workshop consisting of 12 weekly sessions of 3.5 h each; and (2) a control group—the 28 members of this group did not take part in the workshop. 

### 2.3. Data Collection

**Figure 1:**
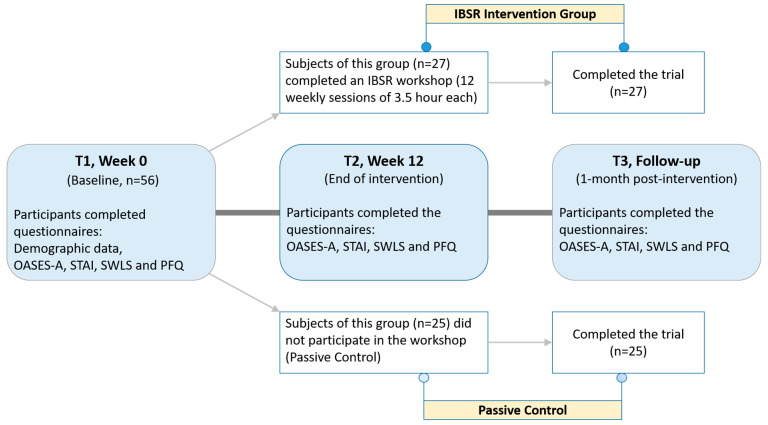
Study timeline. Participants were profiled at baseline (**T1**) for their overall stuttering experience and other psychological indicators. They were then randomly assigned to IBSR or control groups and participated in either a 12-week IBSR intervention program or a 12-week passive control group. Psychological indicators and overall stuttering experience were assessed again at study-week 12 (**T2**) and 1 month after the end of the intervention (**T3**). IBSR = Inquiry Based Stress reduction. OASES-A = Overall Assessment of the Speaker’s Experience of Stuttering-Adults. STAI = State–Trait Anxiety Inventory. PFQ = Psychological Flexibility Questionnaire. SWLS = Satisfaction-with-Life Scale.

This trial was approved by the ethics committee of Tel-Aviv University and has been performed in accordance with the relevant guidelines and regulations (study protocol approval TAU10-2017, registered at 1/11/2017, for consort checklist and trial study protocol, see Supplementary Materials Protocol S1). All trial participants (both the control and intervention groups) completed electronic questionnaires at three different time-points: before the workshop (T1), immediately after the workshop (T2), and one month after the workshop (T3) (Figure 1). Written informed consent and demographic data were obtained from each study participant prior to randomization at T1. Study coordinator (O.F.) generated the random allocation sequence by using the GraphPad Prism randomization tool (simple randomization) in a 1:1 ratio, enrolled the participants, collected baseline questionnaires and assigned participants to either intervention or control group.

### 2.4. Intervention Method

The IBSR intervention involved weekly group meetings (3.5 h/meeting) for 12 weeks. All the sessions were standardized according to a training manual and assessed to maintain consistency in the program, that was presented to the trial participants in the name “*Ledaber Mahalev*” (meaning in Hebrew “speaking from the heart”, instructed by E.G.). The intervention program was conducted by two successive workshops, with 14 participants in each workshop.

IBSR is a technique that involves three steps: In Step 1, Participants identify stressful thoughts and write these thoughts down on paper. The main tool for this task is the “Judge-Your-Neighbor” worksheet (Supplementary Materials Worksheet S1). In Step 2, participants inquire into stressful thoughts. Participants, on their own, or with the help of a "facilitator" (a person trained in the IBSR technique), investigate each written thought, one at a time, using a set of four guided questions: (1) Is it true? (2) Can I absolutely know that it is true? (3) How do I react when I believe that thought? and (4) Who would I be without the thought? The self-investigation enables the participant to question their instinctive beliefs and examine their emotional and physical responses during stress evoking situations. This stage is meditative, and the participants are encouraged to allow space to identify their own true answers to the four questions with no predefined agenda. The recommendations are to achieve a state of witnessing awareness, in which a person observes the thoughts that come into their mind without trying to control or direct them. The goal is realization, not rationalization. In Step 3, participants “turn around” their stressful thoughts and attempt to identify possible evidence for the opposite of the thought [34]. 

For example, if the stressful thought is “My boss does not appreciate me”, the participant would ask himself/herself the four questions and explore possible turnarounds such as: turnaround to the self—“I don’t appreciate myself”; turnaround to the other—“I don’t appreciate my boss”; and turnaround to the opposite—“My boss does appreciate me”. The participants are asked to produce three genuine examples in which the turnaround is as true as the original thought. In this way, the participants try cognitive reframing and may discover that there are other possible interpretations that allow them to interpret the world through a less stressful lens. During the program, the participants are also requested to practice, at home, self-inquiry alone or with a partner, in order to internalize the method and practice the insights learned in the workshop. Homework included: Filling the “Judge-Your-Neighbor” worksheet (Supplementary Materials Worksheet S1) on stressful situations, questioning a thought from the worksheet, and finding more examples of turnarounds, (for further details see van Rhijn, 2015) [33]. As a result of this training, it becomes possible to experience situations that were previously perceived as stressful with peace of mind and connectedness [34,41,42].

### 2.5. Instruments and Outcome Measures

The change in a speaker’s experience of stuttering before and after the intervention was defined as the primary outcome for this clinical trial. Secondary outcomes included psychological indicators of anxiety, psychological flexibility, and satisfaction with life. The outcomes for this trial were assessed using the following validated questionnaires: 

#### 2.5.1. Overall Assessment of the Speaker’s Experience of Stuttering-Adults (OASES-A)

The purpose of the OASES-A questionnaire is to assess the overall stuttering experience of adults who stutter, and quantify the quality of life, satisfaction and overall personal experience of an adult coping on a daily basis with stuttering. The self-administered questionnaire, consists of 100 items, with each item rated on a 5-point Likert scale. The items in the questionnaire are divided into four main sections: (1) General information on stuttering awareness and perception contains 20 items that reflect the knowledge and feelings of the speaker about stuttering in general and, in particular, about his/her own stuttering and also reflect his/her knowledge about existing stuttering treatment techniques. For example, it contains items such as “How do you feel about how you sound when you speak?” or “How knowledgeable are you about factors that affect stuttering”. (2) Reactions to stuttering contains 30 items describing emotional sensations, behavioral consequences, and cognitive responses that are caused by stuttering or by the thought about it. For example, it contains items such as “How often do you experience physical tension when stuttering?” or “When you think about stuttering, how often do you feel ashamed?”. (3) Communication in daily situations contains 25 items weighing the degree of difficulty that speakers experience in their daily life, when communicating in general situations, at work, in social situations, or with their family at home. This section contains items such as, for example, “How difficult is it for you to talk while under time pressure?” or “How difficult is it for you to order food in a restaurant?”. (4) Quality of life contains 25 items detailing how much the speaker’s quality of life is affected by stuttering and its consequences and define the specific fields where it negatively affected. For example, “Overall, how much does stuttering interfere with your intimate relationships?” or “Overall, how much does stuttering interfere with your sense of self-worth or self-esteem?”.

The OASES-A total score is obtained by summing the scores of the four different sections, where each, in the Hebrew version of the questionnaire used in this study, ranges from 1.0 to 5.0. The score rates the severity of the stuttering experience, with 1.0 indicating a mild impact rating and 5.0 a severe impact rating (higher scores represent a more negative impact of stuttering) [28,43].

#### 2.5.2. State-Trait Anxiety Inventory (STAI)

The STAI questionnaire assesses the tendency of adults to experience anxiety and to inspect their sensitivity to anxiety-provoking situations. The questionnaire distinguishes between two scales of anxiety: anxiety as a state and anxiety as a trait. State anxiety is a person’s tendency to experience anxiety in certain situations and at certain times, depending on the context. Trait anxiety is a person’s consistent tendency to experience anxiety frequently. 

STAI is a self-administered questionnaire comprising 40 items (20 items for each scale of anxiety). The state-anxiety scale includes items such as “I feel calm” and “I feel confused”, for example. The trait-anxiety scale includes items such as “I worry too much over something that really doesn’t matter” and “I am content”. Each item is rated on a 4-point Likert scale. After completing the questionnaire, the scores for each item are summed to obtain the total score. Responders receive two separate total scores, one for each of the scales, where the maximum score for each scale is 80 and the minimum score is 20. For both scales, scores between 20 to 40 are considered normal for the general adult population. The higher the score, the higher the anxiety level [44,45].

#### 2.5.3. Psychological Flexibility Questionnaire (PFQ)

The aim of the PFQ questionnaire is to measure the psychological flexibility of the test responder. Psychological flexibility describes a person’s ability to be open, to perceive change as positive, to focus on the present, and to change or persist in behavior according to changes in internal and external circumstances. The questionnaire consists of 20 items rated on a 6-point Likert scale. Each item is associated with one of 5 factors that refers to a significant domain in psychological flexibility. The 5 factors are as follows: (1) positive perception of change (include items such as “When times are hard, even very hard, I am able to remember that there are better times ahead”); (2) characterization of the self as flexible (include items such as “It is easy for me to think of ways of conduct that are very unconventional”); (3) self-characterization as open and innovative (include items such as “It is important to me to learn from each and every person”); (4) a perception of reality as dynamic and changing (include items such as “Reality is never absolute”); and (5) a perception of reality as “multifaceted” (include items such as “I am open to experiencing the different and the exceptional”). The score of the questionnaire is calculated from the mean score of the 20 items. A higher score reflects a higher level of psychological flexibility [46]. 

#### 2.5.4. Satisfaction-with-Life Scale (SWLS) Score

The SWLS questionnaire evaluates the person’s overall satisfaction with life, based on personal criteria that he defines for himself and based on his perception of life. It comprises 5 items that the subjects are required to rate using a 7-point Likert scale, indicating how well they agree or disagree with each item (for example, “In most ways my life is close to my ideal”). The scores of the five items are then summed to generate a total score ranging from 5 to 35, where a higher score indicates greater satisfaction with the subject’s life. A final grade in the range of 30–35 is a very high score, indicating a person with great satisfaction who is very satisfied with his life [47,48]. 

### 2.6. Statistical Analysis

An intention-to-treat analysis approach was implemented for this trial, and therefore all subjects were included in the data processing. Sample size calculation was conducted by using WinPepi software (version 11.65) and was based on the expected change in OASES-A total test score, the main variable of this study. According to the literature [4,49] we hypothesized that at the end of the intervention program (at T2), a 0.5-point gap at this test would be obtained between the intervention group and the control group. Furthermore, after adjustment of at least 15% of dropout rate that was expected in the study [36,38,39] a required sample size of 56 participants was calculated in order to achieve an alpha of 5% and 80% power. Hence, it was determined that each of the control and intervention groups would consist 28 participants. The statistical software SPSS 25 (IBM, New York City) was used for all the data processing within this study. The similarity of baseline (T1) demographic characteristics of participants in the control and intervention groups was assessed by the Chi-squared test and independent-samples *t*-test.

Pearson correlation coefficients were used to examine the correlation between the different instruments at baseline (T1). Two-Way Repeated Measures ANOVAs were used to examine the changes in the mean score over the three different time points and between the intervention groups as well as the interaction between group and time. The dependent variables were study outcomes (OASES-A, STAI, PFQ, and SWLS). The between-subject variable was group and the within-subject variable was differences between OASES-A scores at T1–T2 and T2–T3. Post hoc comparisons were corrected for multiple comparisons using the Sidak correction. 

Due to baseline (T1) differences between the control and the intervention groups in the OASES-A scores, the Two-Way Repeated Measure ANCOVA test was applied for this questionnaire, in order to assess the mean change in results over the different time points in and between the groups. The covariate was OASES-A score at T1 (baseline) and its effect was the within-subject interaction. Due to a disordinal interaction in the RM-ANOVA test for the PFQ questionnaire, we calculated the simple effect per time point, using the independent-samples *t*-test. 

## 3. Results

### 3.1. Study Cohort and Baseline Demographics Characteristics

**Figure 2:**
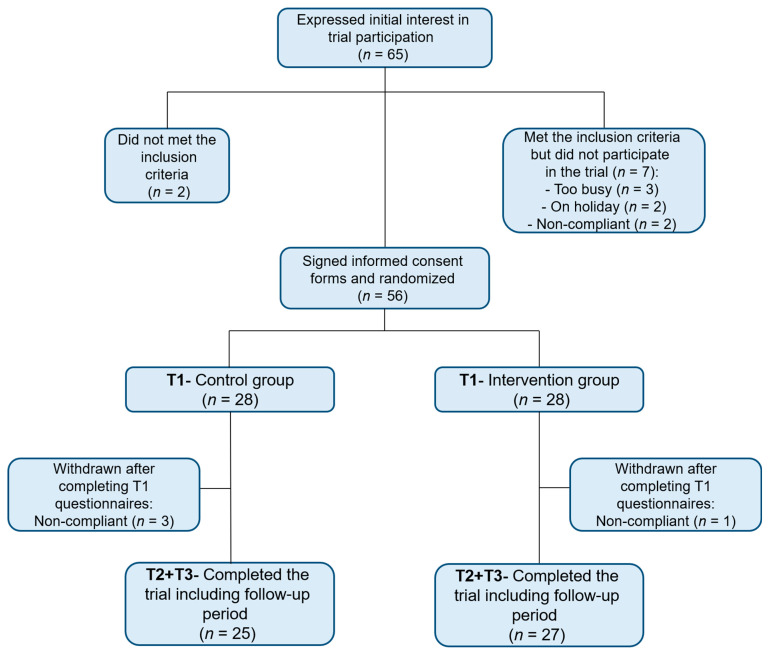
Consort diagram of research flowchart.

Sixty-five Israeli men and women adults who stutter were recruited to this trial between October 2017 and April 2018 (Figure 2). Sixty-three subjects were found eligible to participate in the trial according to the study inclusion and exclusion criteria. Fifty-six participants filled out the initial questionnaires at time point 1 (T1) and were then randomized into two groups: control (*n* = 28) or intervention (*n* = 28). Fifty-two participants completed the questionnaires after completion of the intervention at time point 2 (T2), and at time point 3, one month after the intervention (T3).

Table 1 shows the demographic characteristics of the trial participants. Most of the study participants (80.4%) were males. The mean age of the participants was 33.8 years. All the subjects had completed 12 years of education, and 44.64% had completed tertiary education. According to the collected demographic data, there were no significant differences between the control and the intervention groups.

### 3.2. Overall Stuttering Experience Pre-Post IBSR Intervention

**Figure 3:**
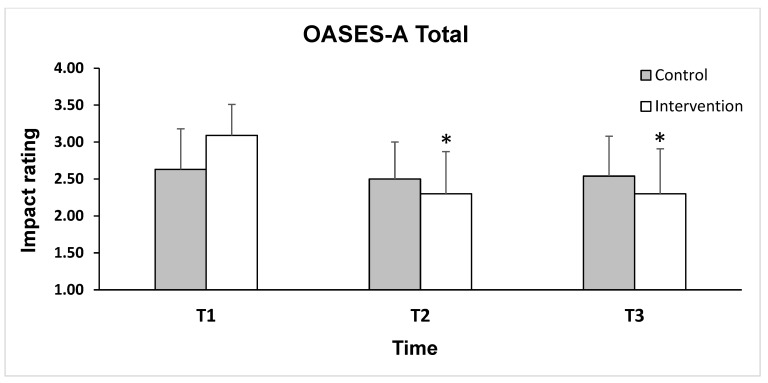
Effect of IBSR intervention on the Overall Assessment of the Speaker’s Experience of Stuttering-Adults (OASES-A) total score in comparison to control group. Data are displayed as mean score ± standard deviation. T1 = before intervention, T2 = after intervention, and T3 = one month after intervention. Impact rating score rates the severity of the stuttering experience: mild (1.00–1.49), mild–moderate )1.50–2.24), moderate (2.25–2.99), moderate–severe (3.00–3.74), and severe (3.75–5.00). * *p* < 0.05.

**Figure 4:**
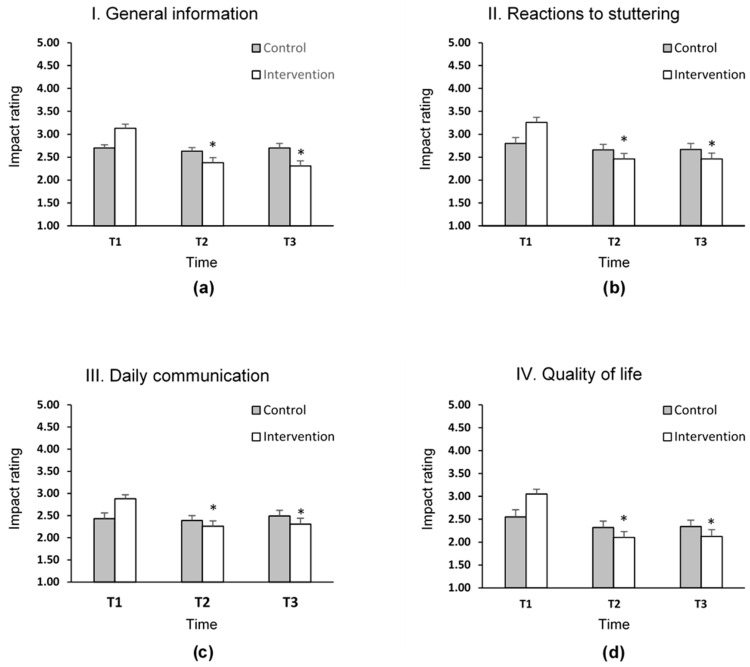
Effect of IBSR intervention on the four sections of Overall Assessment of the Speaker’s Experience of Stuttering-Adults (OASES-A) in comparison to control group. (**a**) OASES-A-I. General information: describes general perspectives about stuttering such as perceived fluency, speech naturalness and knowledge about stuttering (**b**) OASES-A-II. Reactions to stuttering: illustrates speaker’s affective, behavioral and cognitive reactions to stuttering (**c**) OASES-A-III. Daily communication: presents the speaker’s degree of difficulty in various daily communication situations (**d**) OASES-A-IV. Quality of life: describes how much stuttering affects the speaker’s quality of life. Data are displayed as mean score + standard deviation. * *p* < 0.05. T1 = before intervention, T2 = after intervention, and T3 = one month after intervention. Impact rating score rates the severity of the stuttering experience: mild (1.00–1.49), mild–moderate )1.50–2.24), moderate (2.25–2.99), moderate–severe (3.00–3.74), and severe (3.75–5.00).

Table 2 describes the effect of the IBSR intervention on the overall experience of stuttering among AWS. According to the data collected within our trial, there was a marked improvement in the speaker’s experience of stuttering in the intervention group. In this group, the OASES-A total score dropped significantly from 3.09 ± 0.42 at T1 to 2.30 ± 0.57 at T2 (mean difference of 0.78 ± 0.09, *p* < 0.001; Figure 3). The score for the intervention group at T3 remained significantly lower than at T1 (mean difference of 0.78 ± 0.10, *p* < 0.001). The significant mean reduction in scores from T1 to T2 and from T1 to T3 in the intervention group was apparent in all sections of the OASES-A questionnaire: general information, reactions to stuttering, daily communication, and quality of life (Figure 4). In contrast, no significant difference between the three time points was observed in the control group, neither in the OASES-A total score, nor in any of the four components of the questionnaire. Moreover, as shown in Figure 3, we found a significant change between the OASES-A score in the intervention group over the time points in comparison to the control (time **×** group interaction test, *p* = 0.001).

### 3.3. Psychological Indicators among Adults Who Stutter-Pre-Post IBSR Intervention

The trait-anxiety mean score (STAI_B questionnaire, Table 3) of participants from the intervention group decreased significantly from 43.11 ± 8.44 at T1 to 35.89 ± 8.09 at T2 (mean difference of 7.22 ± 1.23, *p* < 0.001) and remained significantly lower than baseline (T1) at T3 (mean difference of 6.78, *p* < 0.001). Compared to the intervention group, no significant change in trait-anxiety level mean score of the control group was observed at any of the three time points measured. The improvement over time in the intervention group, the Trait Anxiety Inventory (STAI_B), compared to the control group, is emphasized by the time * group interaction test (*p* < 0.001). It should be noted that, in the first part of this questionnaire, the State Anxiety Inventory (STAI_A), there was a decrease in the mean scores between the time points in the intervention group, but unlike the STAI_B results, it was not statistically significant.

Table 3 presents the results of the Psychological Flexibility Questionnaire (PFQ), which exhibited an increase in the mean score of the intervention group at time points T2 (4.97 ± 0.64) and T3 (4.96 ± 0.75) compared to values at T1 (4.80 ± 0.51); however, these differences were not significant. 

Finally, we assessed the effect of IBSR on satisfaction with life (SWLS questionnaire, Table 3). The mean score for the intervention group increased significantly from T1 to T2 (mean difference of 3.96 ± 0.81, *p* < 0.001) and from T1 to T3 (mean difference of 4.59 ± 0.99, *p* < 0.001). The significant increase in satisfaction with life over time of the IBSR group (as opposed to the control group) was also demonstrated by a Two-Way RM-ANOVA interaction (time * group) test (*p* < 0.001). 

### 3.4. Correlation-Analysis of the Trial Measures

Significant positive correlations (Supplementary Materials Table S1) were found between the trait-anxiety (STAI_B) variable and the following measures: reactions to stuttering (OASES-A-II; r = 0.559, *p* < 0.001), quality of life (OASES-A-IV; r = 0.610, *p* < 0.001), overall stuttering experience (OASES-A total; r = 0.588, *p* < 0.001), and daily communication (OASES-A-III; r = 0.411, *p* = 0.002) variables. A significant positive correlation was also seen between quality of life variable (OASES-A-IV) and the state-anxiety variable (STAI_A; r = 0.442, *p* = 0.001). 

Significant negative correlations were found between the Satisfaction-with-Life Scale (SWLS) score and trait-anxiety (STAI_B, r = −0.516, *p* < 0.001) variables, and with quality of life variable (OASES-A-IV; r = −0.425, *p* = 0.001). A significant negative correlation was also demonstrated between psychological flexibility (PFQ) and state-anxiety (STAI_A; r = −0.409, *p* = 0.002) variables.

### 3.5. Internal Consistency of the Trial Instruments

Cronbach’s Alpha coefficients were calculated independently for each of the instruments of our trial (Supplementary Materials Table S2). A good internal consistency was revealed (α ranged between 0.755 to 0.947), indicating a relatively high degree of probability that the items within each instrument were addressing the same constructs. 

## 4. Discussion

The results of the present study demonstrate that IBSR is a beneficial tool for improving the overall stuttering experience and psychological measures in AWS. This clinical trial was the first to examine the effectiveness of an IBSR intervention program among adults who stutter and met the primary end point with a significant improvement in the OASES-A total impact rating score in the intervention group compared to the control group. In addition, there was a significant reduction in trait anxiety (STAI_B) and an increase in satisfaction with life (SWLS) in the AWS in the intervention group following workshop participation. 

OASES is currently the gold standard for assessing general information about speech, reactions to stuttering, the level of daily communication, and the quality of life of people who stutter [28,29]. Our study demonstrated that a significant improvement in OASES scores was achieved through IBSR technique. Processes learned and practiced during the workshop (self-inquiry, internal investigation of thoughts, cognitive reframing, and changing the perception of environment and self to a more positive one) appear to empower people who stutter to live a better quality of life, with a heightened sense of wholeness and self-acceptance, and increased ability to cope with diverse situations.

The impact rating scores of the overall stuttering experience were significantly lower for participants in the IBSR intervention group than in the control group immediately after the workshop (T2), and in the follow-up period (T3). Plexico and colleagues (2005) argued that an essential part of dealing with stuttering is the way in which an individual experiences and feels his own stutter. They observed that individuals who reported difficulties in dealing with the consequences of their stuttering, also experienced anxiety, helplessness, hopelessness, and feelings of low self-esteem. In contrast, those who felt that they cope well with stuttering described themselves and their lives more optimistically and positively [16]. The improvement in stuttering experience we observed following IBSR intervention may contribute to the reduction of negative feelings and enable AWS to cope with stuttering and its consequences.

Similar findings were reported by Beilby and colleagues (2012) who investigated the effect of the psychological–behavioral intervention, ACT, on adults who stutter. Their results demonstrated an improvement in the OASES instrument and major reductions in the adverse impact of stuttering on the lives of the participants, following participation in the program [50]. This reinforces the importance of mind–body and psychological intervention programs and their positive impact on people who stutter. 

Improving awareness is an important component of a number of meditative practices, for example, in MBSR, which combines mindfulness-based meditation with yoga and was designed to increase awareness and reduce stress. According to Boyle (2011), MBSR enhanced awareness levels and attention to thoughts, feelings, and behaviors associated with stuttering, while inhibiting the tendency for denial, and could reduce stuttering relapses [27]. Awareness is beneficial to self-acceptance of the AWS and contributes to acceptance of the stuttering [1,3,8,17,18,19]. The IBSR intervention used in our trial also emphasizes awareness as one of the key components. IBSR is designed to raise awareness to automatic thoughts that cause suffering, to observe the attachment to these thoughts, and to internally inquire (similar to Socratic questioning), whether these thoughts are indeed true. In our trial, following the IBSR intervention, there was a marked improvement in “general information” (Section I in OASES-A instrument), in which the AWS record what they feel and know about their own speech. The increase in Section I of the OASES-A instrument among the IBSR intervention participants implies an improvement in the level of knowledge about treatment options and self-help, in the awareness of how often they can speak fluently without stuttering, and in accepting their feelings about being identified by other people as people who stutters. 

Koedoot et al (2011) described that AWS often experience adverse reactions to stuttering, which may significantly limit their ability to participate in daily activities [2]. In our study, we found that there was an improvement in reactions to stuttering after participating in the workshop. These reactions are mainly expressed in the emotional, behavioral, physiological, and cognitive responses of AWS toward their stuttering. In our study, the intervention group participants exhibited a significant improvement in the “reactions to stuttering” (Section II in OASES-A instrument), with an apparent reduction in negative feelings of anger, frustration, shame, guilt, helplessness, among others. Moreover, completing the workshop, as reflected from our trial results, might well be associated with a lessening of adverse physical sensations during stuttering (for example, reduction in physical tension, facial grimaces, and eye blinking). The increase in the Section II of the OASES-A instrument among the IBSR intervention participants implies less avoidance of speaking in situations in which the individual wants to talk but refrains due to fear of stuttering. In addition, emphasizing this point, our study found a significant correlation between reactions to stuttering and trait anxiety (r = 0.559, *p* < 0.001); therefore, it is possible that an improvement in reactions to stuttering may reduce anxiety in AWS. 

People who stutter often express concern over and fear of verbal-interaction initiatives with the environment and thus may tend to avoid social communication in daily situation [3]. Reducing this tendency and increasing social involvement among AWS is a major objective of psychological treatment techniques, such as CBT, inter alia, that aim to neutralize the ineffective negative thoughts that appear in social situations [14,23]. Our results indicate that IBSR workshop participants exhibited a significant improvement in the element of “communication in daily situations”—Section III in the OASES-A instrument. This measurement reflects improvement of participants’ ability to talk “one-on-one”, with other people on the phone, with a spouse or family member, or to speak to clients or a supervisor in work.

Studies have highlighted a number of particularly vulnerable areas for AWS [2,51,52]. For example, stuttering may have negative effects on education and professional performance [53]. Klein and Hood [6] reported that stuttering is perceived to be a career limiting factor by people who stutter. In addition, stuttering is perceived by AWS, as negatively affecting their marital and family life [54]. Other areas where stuttering can adversely affect the quality of life are human vitality, social and emotional functioning, and mental health. Several mindfulness and guided imagery interventions have been shown to be capable of improving quality of life, mood and reducing stress among people who stutter [1,17,18,19]. In our study, the IBSR intervention led to a significant improvement in the “quality of life” aspect—Section IV in the OASES-A instrument. Stuttering was perceived by the participants from the intervention group as less disruptive in relationships with family, friends, and romantic interactions. In addition, participants perceived themselves as less limited in workplace performance and academic advancement options.

Our study showed positive correlation between trait anxiety and reactions to stuttering (r = 0.589, *p* < 0.001) and overall stuttering experience (r = 0.588, *p* < 0.001). Similar findings of an association between stuttering and anxiety have been widely described and studied in the literature. Some of the psychological adverse symptoms associated with stuttering are manifested by high levels of chronic anxiety, particularly social anxiety [3,7,8]. Experiencing chronic anxiety and stress may harm an individual’s general health and give rise to adverse consequences. It may also result in behavioral changes that have been linked to physical and mental illnesses (for example, depression and strokes) and furthermore, anxiety may further exacerbate stuttering [55]. Anxiety is common in situations that are individually perceived as challenging, stressful, and not fully controllable. Thus, re-evaluation and modification of cognitive perceptions are expected to reduce stress and anxiety [56]. One of the main strengths of the IBSR technique is that it enables the participants to reassess their stressful thoughts regarding their ability to cope with future events, and to change their perceptions about their self-worth and their capacity to overcome difficulties [34]. This process may help them to deal with and reduce subjective feelings of stress, fear, and anxiety. Several recent studies have shown that the IBSR meditation technique helped in reducing state and trait anxiety in various populations. Krispenz and Dickhäuser et al. presented the effectiveness of an IBSR intervention in reducing students’ academic procrastination and test anxiety [57,58]. Another research study has demonstrated the ability of IBSR method to reduce chronic stress and trait anxiety in a general (non-clinical) population [56]. Other studies conducted with the IBSR intervention technique that examined its effect on anxiety in the general population reported a significant improvement in trait anxiety as part of the STAI instrument [37,56,59]. It is evident from our results that the IBSR technique similarly significantly improved the component of the Trait Anxiety Inventory (STAI_B) in AWS who participated in the workshop. The increase in the STAI_B instrument among the IBSR intervention participants implies an improvement in their sense of security, relaxation, calmness, with more ability to overcome accumulating difficulties. In addition, the improvements observed following participation in the workshop may be indirectly associated to beneficial behavioral changes, which can prevent physical and mental health deterioration, that could be caused by anxiety.

In addition to the normative regular everyday life challenges, AWS are faced with the adverse behavioral and cognitive consequences that result from stuttering. As a result, they may experience another potential stress-factor in their lives that could impact and lower satisfaction with life. Satisfaction with life is the result of a subjective appraisal which can lead to a negative or positive sense of well-being and is linked to the ability to manage stress. In our study, satisfaction with life also was found to be negatively correlated to trait anxiety (r = −0.516, *p* < 0.001). In order to be satisfied with life, it is helpful if events are viewed as manageable and in a positive light [5]. This study investigated whether the AWS population, with their increased difficulties in daily living, would demonstrate an improvement in satisfaction with life (SWLS) after an IBSR intervention. As part of the IBSR workshop, the participants learned how to re-evaluate thoughts and how to use this process to achieve a sense of management and control in everyday situations. Our results indicate that, indeed, satisfaction with life improved significantly in participants who learned and practiced the IBSR technique, and they were subsequently able to apply the tools they learned to their daily activities. These finding are in concurrence with previous studies that reported a positive effect of IBSR on satisfaction with life in a general population of adults [33], and other studies that assessed the effect of meditation practice on satisfaction with life in a myriad of populations [60]. 

The results of our study suggest that the levels of psychological flexibility (PFQ) did not differ between groups. Participants entered the study at T1 phase (baseline) with fairly high level of psychological flexibility [61,62], and this might create a “floor effect”, which reduces the chances of improvement. 

This study strength includes its randomized controlled design, high compliance levels, and follow up assessments after the end of the IBSR intervention, however, we are aware of several limitations. First, 80% of the sample were male AWS. Although this proportion is similar to gender variance in stutter population, larger female AWS samples would be required to generalize the finding to both genders. Second, study outcomes were based on subjective outcomes that may be affected by response bias. Although the randomized control design may minimize this effect, future studies should also assess the effect of IBSR on objective outcomes, such as fluency of speech, and the timing and frequency of stuttering. Third, we used a non-treatment control group design which is susceptible to non-specific effects (e.g., attention and group support). Lastly, the results indicated that the effect of the intervention was maintained over the month follow-up period. A longer duration of follow-up would be needed to assess whether the improvements of the study outcomes are maintained in the long term. This assessment is important since IBSR can be practiced alone or with others and does not require a trained facilitator and given the chronic nature of stuttering it is of interest to explore whether improvements gained with IBSR are also retained. 

## 5. Conclusions

This study was the first clinical trial to examine the efficacy of an IBSR intervention in a population of people who stutter. This population often suffers from impaired quality of life, difficulties with communication in daily situations, and feelings of anxiety. The results of this randomized controlled trial demonstrated that a 12-week IBSR workshop improves measures of a speaker’s experience of stuttering (OASES-A total and all its 4 components—general information on stuttering awareness and perception, reactions to stuttering, communication in daily situations, and quality of life), trait-anxiety (STAI_B), and satisfaction with life (SWLS). This positive effect following the intervention was maintained throughout the follow-up period of the study, for one month after the intervention. 

Future studies are warranted to assess the effect of IBSR on fluency of speech, as well as the timing and frequency of stuttering, in order to investigate whether meditative therapy is also capable of improving the physiological aspects of stuttering. 

## Figures and Tables

**Table 1:** Demographic characteristics of the participants (*N* = 56).

Characteristic		Study Population *N* (%) or Mean (SD)	Intervention Group *N* (%) or Mean (SD)	Control Group *N* (%) or Mean (SD)	Difference between Groups		
					Test Statistic	df	*p*-Value
Gender	Female	11 (19.6%)	6 (21.40%)	5 (17.90%)	χ2 = 0.113	1	0.737
	Male	45 (80.4%)	22 (78.60%)	23 (82.10%)			
Age	---	33.8 (12.5)	34.1 (11.9)	33.6 (13.2)	*t*-test for independent	54	0.87
					samples *t* =		(tailed 2)
					−0.165		
Age Onset Stuttering	---	5 (3)	5 (3)	4 (4)	*t*-test for independent	54	0.132
					samples *t* =		(tailed 2)
					−1.529		
Classification	Developmental	21 (63.6%)	13 (72.20%)	8 (53.30%)	χ2 = 1.262	1	0.261
	Acquired	12 (36.4%)	5 (27.80%)	7 (46.70%)			
Country of Origin	Israel	47 (83.9%)	23 (82.10%)	24 (85.70%)	χ2 = 0.132	1	0.716
	Others	9 (16.1%)	5 (17.90%)	4 (14.30)			
Mother tongue	Hebrew	45 (80.4%)	22 (78.60%)	23 (82.10%)	χ2 = 0.113	1	0.737
	Others	11 (19.6%)	6 (21.40%)	5 (17.90%)			
Religion	Jew	55 (98.2%)	27 (96.40%)	28 (100%)	χ2 = 1.018	1	0.313
	Muslim	1 (1.8%)	1 (3.60%)	0 (0%)			
Marital Status	Single	32 (57.1%)	13 (46.40%)	19 (67.90%)	χ2 = 5.925	3	0.115
	Married	20 (35.7%)	12 (42.90%)	8 (28.60%)			
	Divorced	3 (5.4%)	3 (10.70%)	0 (0%)			
	Widow/er	1 (1.8%)	0 (0%)	1 (3.60%)			
Years of education	---	14 (2)	13 (2)	14 (2)	*t*-test for independent	54	0.795
					samples *t* = 0.261		(tailed 2)
Employment status	Unemployed	5 (8.9%)	2 (7.10%)	3 (10.70%)	χ2 = 3.978	4	0.409
	Employed	36 (64.3%)	19 (67.90%)	17 (60.70%)			
	Freelance	6 (10.7%)	4 (14.30%)	2 (7.10%)			
	Housewife	1 (1.8%)	1 (3.60%)	0 (0%)			
	Other	8 (14.3%)	2 (7.10%)	6 (21.40%)			
Experience in meditation	No	40 (71.4%)	22 (78.60%)	18 (64.30%)	χ2 = 1.400	1	0.237
	Yes	16 (28.6%)	6 (21.40%)	10 (35.70%)			
Comorbidity *	No	47 (83.9%)	25 (89.30%)	22 (78.60%)	χ2 = 1.191	1	0.275
	Yes	9 (16.1%)	3 (10.70%)	6 (21.40%			

SD, standard deviation; df, degrees of freedom. * Hypercholesterolemia, asthma, epilepsy, ulcerative colitis, hypertension, thyroid disorders.

**Table 2:** Overall Assessment of the Speaker’s Experience of Stuttering-Adults—comparing IBSR intervention with the control group.

OASES-A Section	Time	Group						Time Factor (within Groups)	Time × Group Interaction (between Groups) *
		Control			Intervention				
		Mean	SD	*p*-Value	Mean	SD	*p*-Value	*p*-Value	*p*-Value
I. General Information	T1	2.7	0.07	-	3.13	0.09	-		
	T2	2.63	0.08	-	2.38	0.11	-	----	----
	T3	2.7	0.1	-	2.31	0.11	-		
	T1–T2	0.06	0.06	0.641	0.75	0.09	<0.001	<0.001	<0.001
	T1–T3	0	0.07	1	0.82	0.1	<0.001	(0.437 **)	(0.404 **)
	T2–T3	−0.06	0.05	0.491	0.07	0.07	0.683		
II. Reactions to Stuttering	T1	2.8	0.13	-	3.26	0.11	-		
	T2	2.66	0.12	-	2.46	0.12	-	----	----
	T3	2.67	0.13	-	2.46	0.13	-		
	T1–T2	0.14	0.06	0.072	0.8	0.11	<0.001	<0.001	<0.001
	T1–T3	0.13	0.06	0.076	0.8	0.11	<0.001	(0.471 **)	(0.311 **)
	T2–T3	−0.01	0.05	0.999	0	0.07	1		
III. Daily Communication	T1	2.43	0.13	-	2.88	0.09	-		
	T2	2.39	0.11	-	2.26	0.12	-	----	----
	T3	2.49	0.13	-	2.31	0.13	-		
	T1–T2	0.03	0.06	0.943	0.61	0.1	<0.001	<0.001	<0.001
	T1–T3	−0.06	0.06	0.7	0.57	0.11	<0.001	(0.269 **)	(0.283 **)
	T2–T3	−0.1	0.04	0.111	−0.04	0.05	0.821		
IV. Quality of Life	T1	2.55	0.16	-	3.05	0.11	-		
	T2	2.32	0.14	-	2.1	0.13	-	----	----
	T3 T1–T2	2.34 0.23	0.14 0.11	0.109	2.12 0.95	0.15 0.12	- <0.001	<0.001	<0.001
	T1–T3	0.21	0.08	0.056	0.93	0.13	<0.001	(0.482 **)	(0.261 **)
	T2–T3	−0.02	0.05	0.969	−0.02	0.06	0.98		
Total Score	T1	2.63	0.55	-	3.09	0.42	-		
	T2	2.5	0.5	-	2.3	0.57	-	----	----
	T3	2.54	0.54	-	2.3	0.61	-		
	T1–T2	0.13	0.05	0.089	0.78	0.09	<0.001	0.358	0.001
	T1–T3	0.08	0.05	0.262	0.78	0.1	<0.001	(0.017 **)	(0.201 **)
	T2–T3	−0.04	0.03	0.34	0	0.05	1		

OASES-A = Overall Assessment of the Speaker’s Experience of Stuttering-Adults. * RM-ANOVA/ANCOVA = Two-Way Repeated Measures ANOVA/ANCOVA. ** Partial Eta Squared (Effect Size).

**Table 3:** Psychological outcome measures—comparing IBSR intervention with control group.

Instrument	Time	Group						Time Factor (Within Groups) *	Time ×
									Group Interaction (between Groups) *
		Control			Intervention				
		Mean	SD	*p*-Value	Mean	SD	*p*-Value	*p*-Value	*p*-Value
State Anxiety Inventory (STAI_A)	T1	38.24	9.56	-	37.89	12.16	-		
	T2	38.2	8.6	-	32.33	8.14	-	----	----
	T3	37.72	11.06	-	33.3	11.34	-		
	T1–T2	0.04	1.82	1	5.56	2.31	0.07	0.099	0.136
	T1–T3	0.52	1.85	0.99	4.59	2.46	0.204	(0.047 ^#^)	(0.040 ^#^)
	T2–T3	0.48	1.57	0.987	−0.96	1.57	0.906		
Trait Anxiety Inventory	T1	40.76	9.8	-	43.11	8.44	-		
(STAI_B)	T2	40.4	8.94	-	35.89	8.09	-	----	----
	T3	38.88	10.08	-	36.33	9.12	-		
	T1–T2	0.36	1.21	0.988	7.22	1.23	<0.001	<0.001	<0.001
	T1–T3	1.88	1.18	0.329	6.78	1.37	<0.001	(0.241 ^#^)	(0.151 ^#^)
	T2–T3	1.52	0.98	0.355	−0.44	1.05	0.966		
Psychological Flexibility Questionnaire (PFQ)	T1	4.82	0.63	-	4.8	0.51	-	0.171 ^a^	0.176 ^b^
	T2	4.71	0.75	-	4.97	0.64	-	0.919 ^a^	−1.366 ^b^
	T3	4.79	0.76	-	4.96	0.75	-	0.959 ^a^	−0.799 ^b^
	T1–T2	0.16	0.08	0.169	−0.18	0.1	0.181		
	T1–T3	0.08	0.09	0.762	−0.17	0.11	0.378	----	----
	T2–T3	−0.08	0.07	0.619	0.01	0.09	0.998		
Satisfaction-with-Life Scale (SWLS)	T1	21.16	6.9	-	20.33	4.48	-		
	T2	20.32	7.27	-	24.3	4.66	-	----	----
	T3	21.76	7.07	-	24.93	4.55	-		
	T1–T2	0.84	0.64	0.491	−3.96	0.81	<0.001	<0.001	<0.001
	T1–T3	−0.60	0.77	0.825	−4.59	0.99	<0.001	(0.186 ^#^)	(0.181 ^#^)
	T2–T3	−1.44	0.67	0.123	−0.63	0.66	0.726		

* RM-ANOVA/ANCOVA—Two-Way Repeated Measures ANOVA/ANCOVA. # Partial Eta Squared (Effect Size). ^a^ The score represents a *t*-value of a *t*-test; *t*-test was used due to a disordinal interaction instead of the RM-ANOVA. ^b^ The score represents a *p*-value of a *t*-test.

## Data Availability

The data presented in this study are available on request from the corresponding author.

## Supplementary Materials

The following are available online at https://www.mdpi.com/article/10.3390/jcm10102187/s1. Worksheet S1: “Judge-Your-Neighbor” worksheet. Table S1: Pearson correlation between measures at baseline (N = 56). Table S2: Cronbach’s Alpha for instruments. CONSORT S1: CONSORT 2010 checklist. Protocol S1: Trial study protocol.
