# Supplementary material for: Inquiry Based Stress Reduction (IBSR) Improves Overall Stuttering Experience among Adults Who Stutter: A Randomized Controlled Trial
Source: J Clin Med. 2021 May 18;10(10):2187. doi: 10.3390/jcm10102187 (PMC8158472; doi:10.3390/jcm10102187)
Supplement: Supplementary file 1 [file jcm-10-02187-s001.zip › jcm-1155404-supplementary.pdf]

Inquiry Based Stress Reduction (IBSR) improves overall stuttering experience among adults who stutter: A randomized controlled trial

Omrit Feldman[1], Eran Goldstien[1], Benjamin Rolnik[2], Ariel B. Ganz[2], Shahar Lev-Ari[1,*]

[1] Department of Health Promotion, School of Public Health, Sackler Faculty of Medicine, Tel-Aviv University, Tel-Aviv 69978, Israel; omritfel@gmail.com; erangold1122@gmail.com
[2] Department of Genetics, Stanford University, Stanford, CA 94305, USA; abganz@stanford.edu; rolnik@stanford.edu

* Correspondence: leva@tauex.tau.ac.il; Tel.: +972-3-6405717

Supplementary Material

Worksheet S1. "Judge-Your-Neighbor" worksheet

Judge-Your-Neighbor Worksheet

Think of a stressful situation with someone—for example, an argument. As you meditate on that specific time and place and begin to feel what that felt like, fill in the blanks below. Use short, simple sentences.

1. In this situation, who angers, confuses, hurts, saddens, or disappoints you, and why?

I am ______ (emotion) with ______ (name) because ______

I am angry with Paul because he lied to me.

WANTS 2. In this situation, how do you want him/her to change? What do you want him/her to do?

I want ______ (name) to ______

I want Paul to see that he is wrong. I want him to stop lying to me.

ADVICE 3. In this situation, what advice would you offer him/her? "He/she should/shouldn't…"

______ (name) should/shouldn't ______

Paul shouldn't frighten me with his behavior. He should take a deep breath.

NEEDS 4. In order for *you* to be happy in this situation, what do you need him/her to think, say, feel, or do?

I need ______ (name) to ______

I need Paul to stop talking over me. I need him to really listen to me.

COMPLAINTS 5. What do you think of him/her in this situation? Make a list. (It's okay to be petty and judgmental.)

______ (name) is ______

Paul is a liar, arrogant, loud, dishonest, and unconscious.

6. What is it about this person and situation that you don't ever want to experience again?

I don't ever want ______

I don't ever want Paul to lie to me again. I don't ever want to be disrespected again.

Now question each of your statements, using the four questions of The Work, below. For the turnaround to statement 6, replace the words *I don't ever want…* with *I am willing to…* and *I look forward to…*

The four questions

Example: Paul lied to me.

1. Is it true? (Yes or no. If no, move to question 3.)
2. Can you absolutely know that it's true? (Yes or no.)
3. How do you react, what happens, when you believe that thought?
4. Who or what would you be without the thought?

Turn the thought around.

I lied to me.

I lied to *Paul*.

Paul *didn't* lie to me.

Paul *told me the truth*.

As you visualize the situation, contemplate how each turnaround is as true or truer.

THE WORK OF BYRON KATIE © 2019 Byron Katie International, Inc. All rights reserved. thework.com

6 Feb. 2019

#.

Table S1. Pearson correlation between measures at baseline (N=56)#

Instrument		OASES-A section I	OASES-A section II	OASES-A section III	OASES-A section IV	OASES-A total	STAI_A	STAI_B	PFQ	SWLS
OASES-A section I	r	1								
	P value	-								
OASES-A section II	r	0.557**	1							
	P value	<0.001	-							
OASES-A section III	r	0.496**	0.643**	1						
	P value	<0.001	<0.001	-						
OASES-A section IV	r	0.516**	0.750**	0.730**	1					
	P value	<0.001	<0.001	<0.001	-					
OASES-A total	r	0.697**	0.900**	0.848**	0.911**	1				
	P value	<0.001	<0.001	<0.001	<0.001					
STAI_A	r	0.166	0.327*	0.396**	0.442**	0.412**	1			
	P value	0.223	0.014	0.003	0.001	0.002	-			
STAI_B	r	0.324*	0.559**	0.411**	0.610**	0.588**	0.697**	1		
	P value	0.015	<0.001	0.002	<0.001	<0.001	<0.001	-		
PFQ	r	-0.296*	-0.082	-0.276*	-0.291*	-0.260	-0.409**	-0.258	1	
	P value	0.027	0.549	0.039	0.030	0.053	0.002	0.055	-	
SWLS	r	-0.216	-0.323*	-0.214	-0.425**	-0.363**	-0.363**	-0.516**	0.295*	1
	P value	0.109	0.015	0.113	0.001	0.006	0.006	<0.001	0.027	-

OASES-A= Overall Assessment of the Speaker's Experience of Stuttering-Adults.

OASES-A: section I =General information, section II= Reactions to stuttering, section III= Daily communication,

section IV= Quality of life.

STAI_A= State Anxiety Inventory. STAI_B= Trait Anxiety Inventory.

PFQ= Psychological Flexibility Questionnaire. SWLS= Satisfaction with Life Scale.

r- Pearson correlation.

* Correlation is significant at the 0.05 level (2-tailed).

** Correlation is significant at the 0.01 level (2-tailed).

Table S2. Cronbach's Alpha for instruments

Instrument	Time	Cronbach's Alpha
**OASES-A General information**	T1	0.877
	T2	0.886
	T3	0.902
**OASES-A Reactions to stuttering**	T1	0.803
	T2	0.855
	T3	0.835
**OASES-A Daily communication**	T1	0.819
	T2	0.880
	T3	0.871
**OASES-A Quality of life**	T1	0.794
	T2	0.861
	T3	0.843
**OASES-A total**	T1	0.865
	T2	0.900
	T3	0.895
**State Anxiety Inventory (STAI_A)**	T1	0.926
	T2	0.888
	T3	0.947
**Trait Anxiety Inventory (STAI_B)**	T1	0.892
	T2	0.892
	T3	0.918
**Psychological Flexibility Questionnaire (PFQ)**	T1	0.873
	T2	0.935
	T3	0.947
**Satisfaction with Life Scale (SWLS)**	T1	0.755
	T2	0.887
	T3	0.875

OASES-A= Overall Assessment of the Speaker's Experience of Stuttering- Adults.

CONSORT S1. CONSORT 2010 checklist of information to include when reporting a randomised trial*

Section/Topic	Item No	Checklist item	Reported on page No
**Title and abstract**			
	1a	Identification as a randomised trial in the title	1
	1b	Structured summary of trial design, methods, results, and conclusions (for specific guidance see CONSORT for abstracts)	1
**Introduction**			
Background and objectives	2a	Scientific background and explanation of rationale	1-2
	2b	Specific objectives or hypotheses	2
**Methods**			
Trial design	3a	Description of trial design (such as parallel, factorial) including allocation ratio	3
	3b	Important changes to methods after trial commencement (such as eligibility criteria), with reasons	NA
Participants	4a	Eligibility criteria for participants	3
	4b	Settings and locations where the data were collected	3
Interventions	5	The interventions for each group with sufficient details to allow replication, including how and when they were actually administered	3-4
Outcomes	6a	Completely defined pre-specified primary and secondary outcome measures, including how and when they were assessed	3-5
	6b	Any changes to trial outcomes after the trial commenced, with reasons	NA
Sample size	7a	How sample size was determined	6
	7b	When applicable, explanation of any interim analyses and stopping guidelines	NA
Randomisation:			
Sequence generation	8a	Method used to generate the random allocation sequence	3
	8b	Type of randomisation; details of any restriction (such as blocking and block size)	3

Allocation concealment mechanism	9	Mechanism used to implement the random allocation sequence (such as sequentially numbered containers), describing any steps taken to conceal the sequence until interventions were assigned	NA
Implementation	10	Who generated the random allocation sequence, who enrolled participants, and who assigned participants to interventions	3
Blinding	11a	If done, who was blinded after assignment to interventions (for example, participants, care providers, those assessing outcomes) and how	NA
	11b	If relevant, description of the similarity of interventions	NA
Statistical methods	12a	Statistical methods used to compare groups for primary and secondary outcomes	6
	12b	Methods for additional analyses, such as subgroup analyses and adjusted analyses	6
**Results**			
Participant flow (a diagram is strongly recommended)	13a	For each group, the numbers of participants who were randomly assigned, received intended treatment, and were analysed for the primary outcome	4, 6-7
	13b	For each group, losses and exclusions after randomisation, together with reasons	7
Recruitment	14a	Dates defining the periods of recruitment and follow-up	6
	14b	Why the trial ended or was stopped	NA
Baseline data	15	A table showing baseline demographic and clinical characteristics for each group	8
Numbers analysed	16	For each group, number of participants (denominator) included in each analysis and whether the analysis was by original assigned groups	6-7
Outcomes and estimation	17a	For each primary and secondary outcome, results for each group, and the estimated effect size and its precision (such as 95% confidence interval)	9-13
	17b	For binary outcomes, presentation of both absolute and relative effect sizes is recommended	NA
Ancillary analyses	18	Results of any other analyses performed, including subgroup analyses and adjusted analyses, distinguishing pre-specified from exploratory	NA
Harms	19	All important harms or unintended effects in each group (for specific guidance see CONSORT for harms)	NA
**Discussion**			

Limitations	20	Trial limitations, addressing sources of potential bias, imprecision, and, if relevant, multiplicity of analyses	16
Generalisability	21	Generalisability (external validity, applicability) of the trial findings	16
Interpretation	22	Interpretation consistent with results, balancing benefits and harms, and considering other relevant evidence	14-16
**Other information**			
Registration	23	Registration number and name of trial registry	3
Protocol	24	Where the full trial protocol can be accessed, if available	3
Funding	25	Sources of funding and other support (such as supply of drugs), role of funders	17

*We strongly recommend reading this statement in conjunction with the CONSORT 2010 Explanation and Elaboration for important clarifications on all the items. If relevant, we also recommend reading CONSORT extensions for cluster randomised trials, non-inferiority and equivalence trials, non-pharmacological treatments, herbal interventions, and pragmatic trials. Additional extensions are forthcoming: for those and for up to date references relevant to this checklist, see www.consort-statement.org.

Protocol S1. Trial study protocol

Unique Protocol ID: IBSR-Stuttering

Brief Title: IBSR Intervention for Adults Who Stutter

Official Title: The Effect of "Inquiry Based Stress Reduction" (IBSR) on the Overall Stuttering Experience, Quality of Life and Psychological Indicators Among Adults Who Stutter a Randomized Controlled Clinical Trial

Date: November 1, 2017

Sponsor: Tel-Aviv Sourasky Medical Center and the Israeli Stuttering Association (AMBI).

Investigators:
Dr. Shahar Lev-Ari, PhD.
Mrs. Omrit Feldman, MPH.

Summary

Stuttering is a speech disorder that can cause disturbances in the timing and flow of speech. It is often accompanied not only by verbal difficulties but also by negative impact on several psycho-social aspects in the life of the people who stutter.

Recently, clinical evidences have shown the effectiveness of Inquiry Based Stress Reduction (IBSR) technique to improve psychosocial symptoms and enhance well-being in clinical and non-clinical samples. IBSR, the clinical application of Byron Katie's "The Work", enables individuals to mindfully spot and investigate in a systematic and comprehensive manner thoughts that lead to stress and suffering by a series of questions and turnarounds. IBSR was found to have significantly positive influence on the quality of life, mental well-being and stress management in a variety of populations which suffer from elevated levels of stress, suffering, tension and anxiety, and to enhance resilience of non-clinical general population.

In our research we hypothesized that:

1. Adults who stutter will have high levels of anxiety, and low levels of psychological flexibility and satisfaction with life.
2. IBSR intervention will improve experience of stuttering in adults who stutter.
3. IBSR will decrease anxiety levels and enhance psychological flexibility and satisfaction with life.

Study Design

Study Type: Interventional (Clinical Trial)
Primary Purpose: Treatment
Interventional Study Model: Parallel Assignment
Number of Arms: 2
Masking: None (Open Label)
Allocation: Randomized
Enrollment: 56 subjects

Arms and Interventions

Arm	Assigned Interventions
Experimental: Inquiry Based Stress Reduction (IBSR) workshop	Behavioral:<br>Participants of this group received an IBSR intervention workshop. The IBSR intervention workshop included weekly group meetings (3.5 hours/meeting) for 12 weeks. During the workshop, participants were encouraged to identify and inquire their stressful thoughts. Using self-inquiry practices participants were taught to increase awareness of their thoughts and feelings, to observe their emotional and physical responses during situations perceived by them as stressful, and allow their mind to return to its true, peaceful, creative nature. Through the process of self-inquiry, participants took an active role in investigating their stressful thoughts, and by this regulated their stress and managed symptoms and emotions, thus enabled them to cope better with the psycho-social consequences of the stuttering.<br>Other Name: "The Work" meditation (Byron Katie)
No Intervention: Control group	None:<br>Participants of this group did not take a part in the workshop.

Outcome Measures

Primary Outcome Measure:

1. Overall Assessment of the Speaker's Experience of Stuttering for Adults (OASES-A) [Time Frame: 1) Before the workshop (T1), 2) Immediately after the workshop (T2), 3) One month after the workshop (T3)].

   The purpose of OASES-A questionnaire is to assess the overall stuttering experience of adults who stutter. It quantifies the quality of life, satisfaction and overall personal experience of the adult in his daily coping with stuttering. The items in the questionnaire are divided into four main sections: 1. General information on stuttering awareness and perception. 2. Reactions to stuttering. 3. Communication in daily situations. 4. Quality of life.

   The OASES-A total score is obtained by summing the scores of the four different sections. In this trial we used the Hebrew version of this questionnaire. OASES-A total score and the score for each of the sections (in the Hebrew version) is ranging from 1.0 to 5.0. The score rates the severity of the stuttering experience, where 1.0 indicates a mild impact rating and 5.0 a severe impact rating (higher scores indicate a more negative impact of stuttering) (Freud et al., 2017; Yaruss & Quesal, 2006).

Secondary Outcome Measures:

1. State-Trait Anxiety Inventory (STAI) [Time Frame: 1) Baseline (Before the workshop (T1)). 2) Immediately after the workshop (T2). 3) One month after the workshop (T3).]

   The STAI questionnaire assess the tendency of adults to experience anxiety and to inspect their sensitivity to anxiety-provoking situations. The questionnaire distinguishes between two scales of anxiety: anxiety as a state and anxiety as a trait. State anxiety is a person's tendency to experience anxiety in certain situations and times, depending on their context. Trait anxiety is a person's constant and stable tendency to experience anxiety frequently.

   Each item is rated on a 4-point Likert scale. After completing the questionnaire, items scores are added to obtain total scores. Responders receives 2 separate total scores, one for each of the scales. The maximum score for each scale is 80 and the minimum score is 20. In both scales, scores between 20 to 40 is considered normal in the general adult population. The higher the score, the higher the anxiety level (Spielberger et al., 1970; Taychman & Malinek, 1984).

2. Psychological Flexibility Questionnaire (PFQ) [Time Frame: 1) Baseline (Before the workshop (T1)). 2) Immediately after the workshop (T2). 3) One month after the workshop (T3).]

   The aim of PFQ questionnaire is to measure the psychological flexibility of the test responder. Psychological flexibility describes a person's ability to be open, to perceive change as positive, to focus on the present and to change or persist in behavior according to changes of internal and external circumstances. The questionnaire consists of 20 items rated on a 6-point Likert scale. Each item is associated with one of 5 factors that refers to a significant domain in psychological flexibility. The 5 factors are: 1. Positive perception of change. 2. Characterization of the self as flexible. 3. Self-characterizing as open and innovative. 4. A perception of reality as dynamic and changing. 5. A perception of reality as "multifaceted". The score of the questionnaire is calculated based on the mean of the 20 items. Higher score indicates high level of psychological flexibility (Ben-Itzhak et al., 2014).

3. Satisfaction with Life Scale (SWLS) [Time Frame: 1) Baseline (Before the workshop (T1)). 2) Immediately after the workshop (T2). 3) One month after the workshop (T3).]

   The SWLS questionnaire evaluates the person's overall satisfaction with life, based on personal criteria that he defined for himself and based on his perception of life. In the questionnaire, there is 5 items that the subjects are required to rate using a 7-point Likert scale. The five items are summed to create a total score that ranged from 5 to 35, with a higher score indicates greater satisfaction with the subject's life. A final grade in the range of 30-35 is a very high score, indicating a person with great satisfaction and very satisfied with his life (Diener, 1994; Diener et al., 1985).

Eligibility Criteria

Ages Eligible for Study: 18 Years and older
Sexes Eligible for Study: All

Inclusion Criteria:

- Diagnosis of either developmental or acquired stuttering.
- Capability to understand and fill out the study outcome instruments and informed consent form.

Exclusion Criteria:

- Diagnosis of severe mental illness.
- Inability to understand or read Hebrew.

- Subjects who declare they cannot take a part in the trial throughout its whole duration.

Contacts/Locations

Central Contact Person: Shahar Lev-Ari, PHD
Email: leva@tauex.tau.ac.il

Central Contact Backup: Omrit Feldman, MPH
Email: omritfel@gmail.com

Study Officials:
Locations: Sackler Faculty of Medicine, Tel Aviv University
Tel Aviv, Israel, 6139001, Israel

References

1. Freud, D., Kichin-Brin, M., Ezrati-Vinacour, R., Roziner, I., & Amir, O. (2017). The relationship between the experience of stuttering and demographic characteristics of adults who stutter. *Journal of Fluency Disorders*. https://doi.org/10.1016/j.jfludis.2017.03.008

2. Yaruss, J. S., & Quesal, R. W. (2006). Overall Assessment of the Speaker's Experience of Stuttering (OASES): Documenting multiple outcomes in stuttering treatment. *Journal of Fluency Disorders*. https://doi.org/10.1016/j.jfludis.2006.02.002

3. Spielberger, C. D., Gorsuch, R. L., & Lushene, R. E. (1970). STAI manual for the state-trait anxiety inventory. Self-Evaluation Questionnaire. In *MANUAL*. https://doi.org/10.1037/t06496-000

4. Taychman, Y., & Malinek, F. (1984). *STAI, STAIC: A questionnaire evaluating state anxiety and trait anxiety- The Hebrew guide for the examiner*. Ramot Ltd., Tel-Aviv University.

5. Ben-Itzhak, S., Bluvstein, I., & Maor, M. (2014). The Psychological Flexibility Questionnaire (PFQ): Development, Reliability and Validity. *WebmedCentral Psychology*. https://doi.org/10.9754/journal.wmc.2014.004606

6. Diener, E. (1994). Assessing subjective well-being: Progress and opportunities. Social Indicators Research. https://doi.org/10.1007/BF01207052

7. Diener, E., Emmons, R. A., Larsem, R. J., & Griffin, S. (1985). The Satisfaction With Life Scale. Journal of Personality Assessment. https://doi.org/10.1207/s15327752jpa4901_13
